# Testing the Tropical Niche Conservatism Hypothesis: Climatic Niche Evolution of *Escallonia* Mutis ex L. F. (Escalloniaceae)

**DOI:** 10.3390/plants13010133

**Published:** 2024-01-03

**Authors:** María José Dibán, Luis Felipe Hinojosa

**Affiliations:** Laboratory of Paleoecology, Department of Ecological Science, Faculty of Science, University of Chile, Santiago 7800003, Chile

**Keywords:** ecological niche modelling, evolutionary models, phylogenetic signal, ancestral area, the Andes

## Abstract

We assess the Tropical Niche Conservatism Hypothesis in the genus *Escallonia* in South America using phylogeny, paleoclimate estimation and current niche modelling. We tested four predictions: (1) the climatic condition where the ancestor of *Escallonia* grew is megathermal; (2) the temperate niche is a derived condition from tropical clades; (3) the most closely related species have a similar current climate niche (conservation of the phylogenetic niche); and (4) there is a range expansion from the northern Andes to high latitudes during warm times. Our phylogenetic hypothesis shows that *Escallonia* originated 52.17 ± 0.85 My, in the early Eocene, with an annual mean temperature of 13.8 °C and annual precipitation of 1081 mm, corresponding to a microthermal to mesothermal climate; the species of the northern and central tropical Andes would be the ancestral ones, and the temperate species evolved between 32 and 20 My in a microthermal climate. The predominant evolutionary models were Brownian and Ornstein–Uhlenbeck. There was phylogenetic signal in 7 of the 9 variables, indicating conservation of the climatic niche. *Escallonia* would have originated in the central and southern Andes and reached the other environments by dispersion.

## 1. Introduction

A broad scale latitudinal gradient of richness of angiosperm families is widely recognized and documented; tropical regions concentrate the highest species diversity, which decreases in temperate zones [1,2,3]. This species distribution pattern is associated with the current climate, primarily temperature and water availability [4], and with historical and biogeographical process [5,6]. The tropical niche conservatism hypothesis (TNCH) [6] proposes that high richness and a high concentration of the oldest angiosperm clades in the tropics [7,8] could be explained by a broad territorial extension for a long time until the middle of the Cenozoic [9], accumulating more species over time. Dispersion to temperate regions was restricted to global warming periods, where species extended their distribution range following their megathermal phylogenetic niche conservatism; then, with the advent cooling periods and high seasonality from the middle of the Cenozoic onwards [10,11], angiosperm clades that developed freezing tolerance could inhabit high latitudes [12]. These would thus be derived and would be more recent than tropical clades [6].

The biogeographic context of South America generated floristic disjunctions in many Neotropical genera such as *Escallonia* Mutis ex L. F., *Azara* Ruiz and Pav., *Crinodendron* Molina, *Myrteola* O. Berg, *Blepharocalyx* O. Berg, *Myrceugenia* O. Berg, *Polylepis* Ruiz and Pav., *Tropaeolum* L. and *Chuquiraga* Juss., among others [13,14]. These are distributed in both tropical and temperate zones; the rise of the Andes and the emergence of the Arid Diagonal are the main factors for the isolation of the forests of South America [15]. Andean orogeny has had an important effect on the diversification and uniqueness of biota [16,17,18], because it produced a geographic barrier for east–west dispersion, isolating organisms through vicariance processes [19]. The Andes also generated an altitudinal gradient that could trigger ecological differentiation and speciation [20]. Several plant lineages associated with the Andes diversified; a few are exclusive to these areas [21], as is the case of *Escallonia*, of the family Escalloniaceae [22].

**Figure 1:**
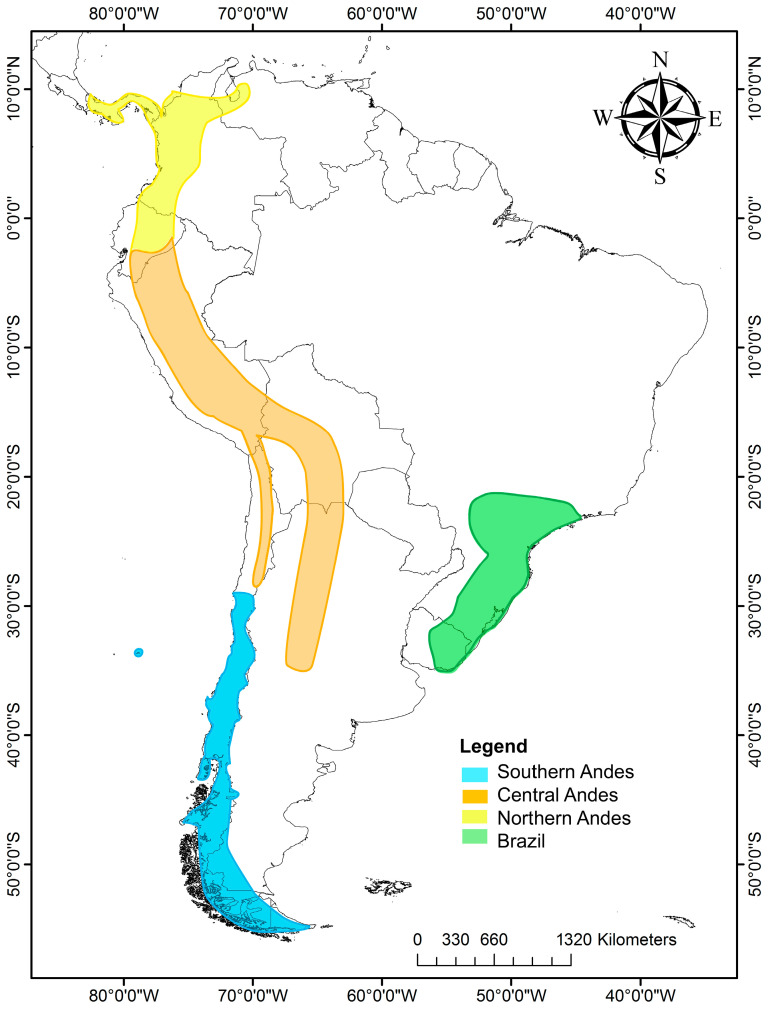
Distribution map of *Escallonia* species in South America. In yellow are shown the species of the northern Andes; in orange, those of the central Andes; in blue, those of the southern Andes; and in green, those of southeastern Brazil and part of Uruguay.

The genus *Escallonia* has 39 species distributed in South America, mostly along the Andes from Panamá to Tierra del Fuego; the northern and central Andes have the highest richness species, followed by Chile, the southern coast of Brazil, Uruguay and Juan Fernández Island [23]. *Escallonia* can be grouped into four zones according to the morphological characteristics and phytogeographic regions [24] (Figure 1): (1) Southern Andes, including mainly central–southern Chile and Chilean and Argentina Patagonia. There are 13 species in this zone. (2) Central Andes, including Andean zones of northern Chile, Peru, Bolivia and Argentina, in addition to Yungas, mostly above 2000 m in altitude, with 11 species. (3) Northern Andes: this belongs to the Páramo region, extending from Venezuela to the Huancabamba Depression, a biogeographic barrier [25] that begins at the Jubones River (at 4° S), considered the southern limit of our northern Andes [26]. There are six species distributed at over 2000 m in altitude in Panama, Colombia and Ecuador. Some species are also distributed in the central Andes from 4° S to 20° S, including Peru and part of Bolivia. (4) Southeast of Brazil, including southeastern Brazil and eastern Uruguay, with nine species of *Escallonia*. It is important to note that some species exhibit a broader distribution range, covering more than one area, such as *Escallonia micrantha* Mattf., *Escallonia pendula* (Ruiz and Pav.) Pers., *Escallonia paniculata* (Ruiz and Pav.) Roem. and Schult., *Escallonia schreiteri* Sleumer, *Escallonia resinosa* (Ruiz and Pav.) Pers., and *Escallonia myrtilloides* L. F., which cover portions of the central and northern Andes. The species that are distributed in southeast Brazil and Uruguay live in warmer and rainy conditions, and the Chilean and Argentine species are in the coldest zones (Table 1).

Most *Escallonia* species live close to fresh water sources, open forests, coastal zones and montane forests [27]. *Escallonia* is a monophyletic genus [22,28,29], but its geographic origin is not clear. 

This genus inhabits tropical, Mediterranean and temperate climates. The following questions arise: (1) where and in which climate did the genus *Escallonia* originate? (2) Based on its current distribution and phylogeny, how did the climate niche of this genus evolve? 

According to the TNCH, we make the following predictions for *Escallonia*: (1) it had megathermal ancestral climatic conditions, that is, an average annual temperature greater than or equal to 22 °C and an average annual precipitation over 549 mm [30]; (2) the temperate niche is a derived condition from tropical clades; (3) the most closely related species have a similar current climate niche (conservation of the phylogenetic niche); and (4) there was a range expansion from the northern Andes to high latitudes during warm periods. The aim of this study is to predict the ancestral climatic conditions and geographical distribution of the genus *Escallonia*, and to investigate by which evolutionary model the climate niche would have changed over time until arriving at the present conditions.

## 2. Results

### 2.1. Phylogenetic Reconstruction

**Figure 2:**
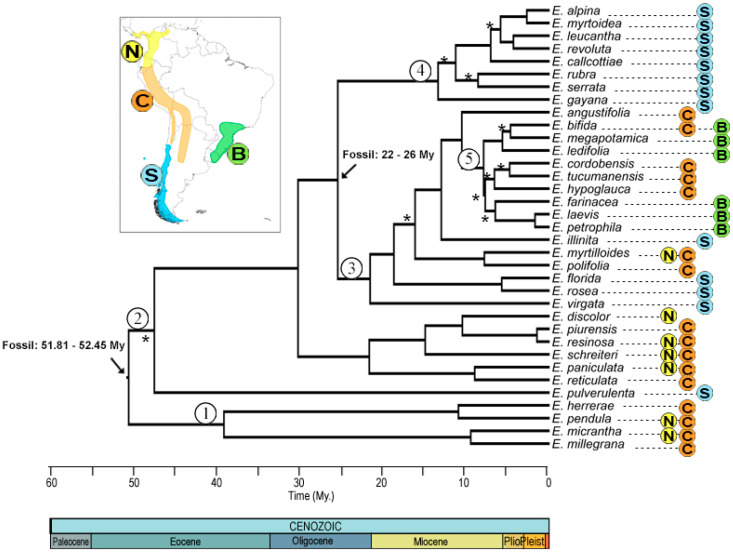
Consensus phylogenetic tree obtained from Bayesian analysis. The distribution of the genus *Escallonia* with the corresponding color nomenclature of each node is shown in the upper left box. The black arrow corresponds to the nodes used to calibrate the phylogeny, with their respective ages and data used. The numbers within a circle correspond to the nomenclature that will be used to name the nodes. Asterisks mark the nodes with a posterior probability of <0.95. Colors and letters indicate the nomenclature of the distribution zones.

Phylogenetic reconstruction by Bayesian inference yielded a consensus tree (Figure 2), in which 10 nodes did not have good statistical support (posterior probability less than 0.95). The most recent common ancestor (MRCA) of the genus dates from 52.17 ± 0.85 My, with divergence of clades 1 and 2 at 40 ± 7 and 46 ± 6 My, respectively, which includes species from the northern Andes and center, except for *E. pulverulenta* from the Southern Andes, but with an unsupported node. Clade 3 has a divergence time of 26.2 ± 3 My, mostly southern Andes with the exception of *E. myrtilloides* and *Escallonia polifolia* Hook. from the northern and central Andes, respectively. Clade 4 includes species from the southern Andes, with a divergence time of 13.7 ± 4 My. Finally, clade 5 groups species from the central Andes and Brazil, with an age of 10.7 ± 2 My.

### 2.2. Ancestral Climate Reconstruction

**Figure 3:**
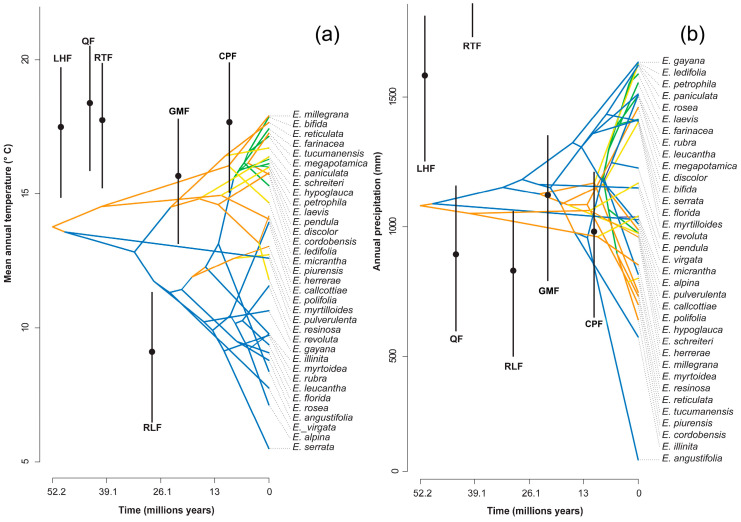
Reconstruction of the climatic niche of (**a**) the mean annual temperature and (**b**) annual precipitation for the genus Escallonia over time. The estimated temperatures for six formations are shown in black: Laguna el Hunco Flora (LHF), Quinamavida Flora (QF), Río Turbio Flora (RTF), Río Leona Flora (RLF), Goterones–Matanzas Flora (GMF), and Cerro Los Pololos Flora (CPF). The points represent the average of climate estimation, and the bars represent the standard error ranges.

The mean annual temperature in which the genus would have originated is 13.8 °C, with an annual precipitation of 1081 mm (Figure 3); this is a micro to mesothermal climate [30]. 

### 2.3. Evolution Models and Niche Conservatism

The most probable climatic evolution models are BM, and only three are OU, which would indicate that there is niche conservation in all climate variables. Furthermore, all except Bio 12 and 16 have phylogenetic signals. According to the results obtained from the evolution models (Table 2), there is conservation of the phylogenetic niche, following a model of gradual change over time (BM), with the exception of Bio 10, Bio 12 and Bio 16, whose evolution models correspond to stabilizing selection (OU). Pagel’s lambda [31] shows that 7 of the 9 variables have a phylogenetic signal (Table 2). Bio 12 and Bio 16’s variables do not have a phylogenetic signal; that is, these climatic variables vary randomly in phylogeny and therefore are independent of the degree of kinship.

### 2.4. Ancestral Area Reconstruction

**Figure 4:**
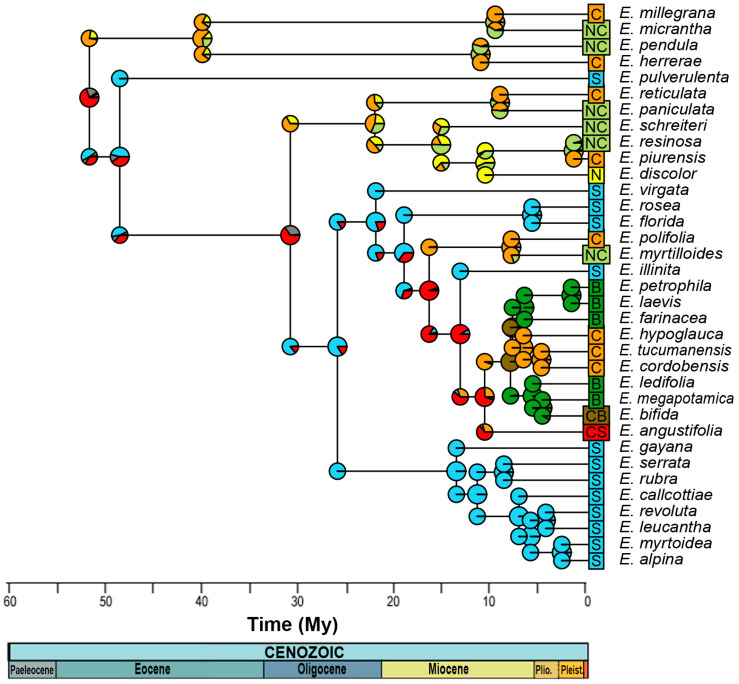
Estimation of the distribution of the most recent common ancestor for each node, according to the DEC model. N corresponds to the northern Andes, Peru being the southern limit; C, the central Andes, including the Yungas and the eastern part of the Cordillera; S, the southern Andes, including south-central Chile; and B, those of Brazil. On the other hand, NC indicates northern and central Andes; CB, central and Brazil; and CS, central and southern Andes. The colors of the circles represent the relative probability of distribution areas, and the mixture between them, such as the brown color, indicates a wider distribution in the central and southern Andes.

For the reconstruction of the ancestral distribution range, the model with the best fit was DEC (LnL = −49.48), with *d* = 0.008, *e* = 0, and *j* = 0 (Figure 4, Table S6). This suggest that the most likely initial distribution would have included the entire central and southern Andes, where species would have coexisted in sympatry, following cladogenesis patterns [32]. After the Eocene Climatic Optimum, temperatures started to decrease and reached a minimum at around 35 My when Antarctic glaciation began, associated with the period of separation between Antarctica and Australia. Thus, at ~31 ± 5 My, a dispersion event occurred from the south to the north, reaching the central zone of the Andes, followed by a new vicariance separating the central and southern zones, which is consistent with [13], leading to a decrease in global ocean temperatures, followed by the eastern Antarctic glaciation and the drop in sea level [33], with the thermal gradient between the tropics and the South Pole being pronounced. In the Patagonian Andes, the presence of the rain shadow effect was documented at 34 My ago [34]. The Andes had already begun to rise by this period, reaching ~1500 m above sea level by ~25 My [35]. Thus, the new habitats at altitude would have provided colder climatic conditions, and would have allowed *Escallonia* to inhabit tropical latitudes, but in mesothermal areas.

During the Miocene between 15 ± 3 My, there was an expansion in the distribution of the clade of the central Andes toward the north, and simultaneously, from the southern Andes to the central Andes, followed by a process of vicariance at 13 ± 3 My, with *Escallonia illinita* C. Presl separating towards the south from the central clade. The main dispersion towards the northern Andes occurs in different clades of the phylogeny, but in the same time range, between 15 and 5 My, except for *Escallonia resinosa* (Ruiz and Pav.) Pers., which is the most recent (0.1 to 3 My). During this period, strong pulses of Andean uplift occurred, reaching 3–4 km in altitude between 15 and 10 My [34,35], as did the separation between Antarctica and South America between 23 and 12 My, which led to the complete glaciation of Antarctica and the decline in global ocean temperatures [33]. This event, together with the emergence of the Humboldt Current, would have influenced the origin of the Atacama Desert [13,36]. 

Finally, at 8 ± 2 My, there was an expansion in range from the central Andes to Brazil, with both zones coexisting in sympatry and later vicariance to the central Andes. This event is subsequent to the marine transgressions that occurred between 15 and 13 My, where it has been proposed that the Argentinean Patagonia and the eastern coasts of Brazil were submerged [37] and could act as a vicariant barrier between the species distributed in Brazil and the central Andes.

## 3. Discussion

### 3.1. Phylogenetic Reconstruction

Phylogenetic reconstruction (Figure 2) indicates that the temperate clades are nested with those of the northern and central Andes. This topology is consistent with one of the predictions of the Tropical Niche Conservation Hypothesis (TNC) [6] which suggests that temperate clades are derived from tropical clades. A similar topology was also recovered by Zapata [22] but included, without support, the Chilean species *E. pulverulenta*. In our analysis, it is grouped in clade 2 (Figure 2), related to the northern and central Andean species and being an ancestor of the rest of the species, but due to the low a posteriori probability (0.52), the position of this species of remains uncertain. However, comparing the leaf and flower morphology of *E. pulverulenta* to the other species of the genus, it is more similar to the species of the basal clade (node 1), which are characterized by having leaves over 7 cm long and inflorescence in a tubular panicle with abundant flowers. The Chilean species have small leaves, most of them averaging 2.5 cm long and reaching a maximum of 6 cm, with panicle inflorescence and with fewer flowers (Figure S1)

### 3.2. Ancestral Climate Reconstruction

Paleoclimatic estimates suggest global temperatures between 8 and 12 °C higher during this period than at present [38], less pronounced equator–pole temperature gradients and tectonic conditions in which South America, Antarctica and Australia were joined, and the Andes did not have its current height [13,39]. The presence of *Escallonia* in the fossil record of Laguna del Hunco (42° S in Argentinean Patagonia) indicates that the genus would have been distributed in the area under mesothermal climate conditions with average annual temperatures of 17.2 ± 2.3 °C and precipitation of 1673 ± 426 mm. [40,41]. Our estimate of 13.8 °C and an annual rainfall of 1000 mm (Figure 3), although slightly lower than 14 °C, is consistent with a mesothermal climate for the early Eocene. These mesothermal conditions would have been widespread in mid (>30° S) and high latitudes of the Paleocene and Eocene in South America [39], which implies that the initial area of diversification of the genus was in temperate latitudes. 

The current distribution of the central–northern Andean species that include clade 1, *E. herrerae*, *E. pendula*, *E. micrantha* and *E. millegrana*, currently inhabit tropical latitudes, but under the conditions of a mesothermal climate (Table S2), from 1669 m above sea level in the Yungas mountain forest (*E. millegrana*) to above 2200 to 2800 m in the central and northern Andes (Table S2), conditions not present during the Eocene. The microthermal conditions observed in our model of mean annual temperature reconstruction (Figure 3) would have arisen during the Oligocene and are consistent with global temperature declines [38] and the southern Patagonia fossil record [34].

### 3.3. Evolution Models and Niche Conservatism

The TNH postulates that lineages would inhabit high latitudes while conserving their climatic niche. Our analysis shows the conservation of niche (Brownian and/or Orstein–Uhlembeck model of evolution (in which the traits evolve constrained by selection towards an optimum, as in the case of stabilizing section [42], Table 2)). When a BM model of evolution operates, it indicates that the difference between the species accumulates over time, because by inheriting the niche from their ancestor, they diverge slowly. When an OU evolution model operates, it indicates that the traits are evolving very slowly in relation to environmental changes, and the species take time to adapt to a new optimum [43]. In addition, a multimodal response is obtained after speciation, where there is only one optimum per branch in the phylogeny [44].

**Figure 5:**
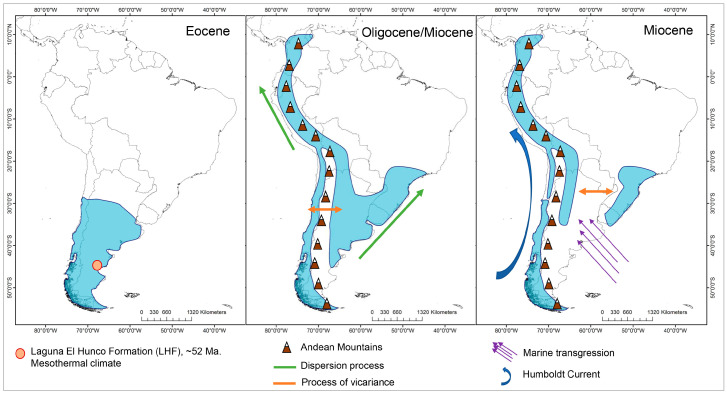
Ancestral area reconstruction of *Escallonia* in South America.

According to the TNH, the tropical clades would have expanded into temperate latitudes during the Eocene, when conditions were warmer. The MRCA in *Escallonia* would have been 52.17 My, the time of the Early Eocene thermal optimum, and our analysis of the distribution of the DEC ancestral area suggests that *Escallonia* would have been continuously distributed from the center to the south of the western margin of South America (Figure 4). This result is not consistent with that previously described by Zapata [22], who suggests that *Escallonia* would have originated in the tropical Andes (central and northern Andes of our analysis, (Figure 1), expanding its distribution southward to include southern Brazil. However, the presence in the fossil record of Laguna del Hunco (Eocene of Argentine Patagonia) of remains of *Escallonia* similar to the clade 1 species (Wilf com pers) suggests an austral origin (Figure 5).

For the above reasons, *Escallonia* would not have originated in tropical latitudes and migrated to temperate zones as proposed by the TNH, but would have followed the opposite pattern, conserving its mesothermal niche, similar to that proposed for *Nothofagus* [45]. The analysis of the distribution of the DEC ancestral area suggests that *Escallonia* would have been distributed in the central and southern zone of the Andes, and that it would have been subsequently segregated between the central and southern zones, ending with the colonization of the northern zone of the Andes and Brazil by means of processes of vicariance and dispersion (range extension).

## 4. Materials and Methods

### 4.1. Niche Modeling

We performed climatic niche modelling with 35 of the 39 species of *Escallonia* using the maximum entropy algorithm, MaxEnt [46]. We selected 9 bioclimatic variables downloaded from the WorldClim database with ≈1 km^2^ resolution [47], using a principal component analysis (PCA). We obtained a minimum of 15 valid occurrences for each species, and a total of 1643 for the genus from GBIF (Global Biodiversity Information Facility) and virtual herbarium records (Neotropical Herbarium Specimens, Herbario Virtual Austral Americano, SEINet: Arizona—New Mexico Chapter, Cooperative Taxonomic Resource for American Myrtaceae, Natural History Museum). For niche modelling, we used 50 replicates for each species, 25% of the data as a training set, a regularization multiplier of 1, and the bootstrap run option. We used AUC values to test if MaxEnt predictions differ from random. To obtain predicted niche occupancy (PNO) for each species [48] we used the “PHYLOCLIM” package of R [49]. We estimated the weighted mean for each of the 9 bioclimatic variables for posterior analysis of ancestral climate reconstruction.

Finally, we used the bioclimatic thermal regime proposed by [30]: megathermal climate (MAT ≥ 22 °C, MAP > 549 mm); mesothermal climate (MAT > 14–22 °C, MAP > 549 mm); and microthermal climate (MAT ≤ 14 °C, MAP 719–3000 mm).

### 4.2. Phylogenetic Reconstruction

We obtained 37 sequences from GenBank (https://www.ncbi.nlm.nih.gov/genbank/, accessed on 5 May 2016) generated by Zapata [22] corresponding to the nuclear NIA gene (anthocyanin regulator). Thirty-five of these sequences correspond to the *Escallonia* species mentioned previously, and the other two are from *Valdivia gayana* (J. Rémy) and *Forgesia racemosa* (J.F. Gmel.), both belonging to the family Escalloniaceae and sister species of *Escallonia*, which were used as an external group (Table S1).

Sequences were aligned with the BioEdit program [50], obtaining a total length of 890 base pairs. Phylogeny was reconstructed by means of Bayesian analysis through the BEAST 2 program [51], using the nucleotide substitution model TIM3 + G (AIC = 8.310.6), and an analysis was carried out by MCMC with 10,000,000 generations. Two calibration points were used with the strict clock model, corresponding to the fossil record of *Escallonia* with affinity to three current Chilean species: *Escallonia rubra* (Ruiz and Pav.) Pers., *Escallonia myrtoidea* Bertero ex DC., and *Escallonia rosea* Griseb., of the Cura-Mallín formation, VII Region [52], dated between 22 and 26 Ma. [53]; and a fossil description not yet published (Wilf, pers. Comm.) assigned to the genus *Escallonia* [54] in the Laguna del Hunco Formation, dated between 51.81 and 52.45 Ma. [40], which was related for this study with *E. micrantha*, *Escallonia millegrana* Griseb., *E. pendula*, *Escallonia herrerae* Mattf., and *Escallonia pulverulenta* (Ruiz and Pav.) Pers. due to morphological similarities. It was found that the parameters reached convergence using the Tracer v1.6 program [55].

Finally, the values obtained from the weighed PNOs were used for the climatic and evolutionary reconstruction of the niche (Table S4). The reconstruction of ancestral characters for each node was performed with the function “phytools” in R [48]; this builds phenograms, which plots a projection of the phylogenetic tree in a space defined by phenotype or a character on the “*y*” axis, and time on the “*x*” axis. In addition, it was compared with the estimated paleoclimate for 5 fossil localities of Chile and Argentina: (1) The fossil flora of Laguna del Hunco is located in the Huitrera Formation, which was dated to ~52 million years ago (My) [40], with a mean annual temperature (MAT) of 17.2 °C, and mean annual precipitation (MAP) of 1973 mm [40]. (2) The Quinamávida fossil flora is situated in the Cura-Mallín Formation, with an age of 46–45 My [53], a MAT of 18.3 °C, and MAP of 888 mm [41]. (3) The Río Turbio Formation and the associated fossil flora are dated to 44.6–34 My [56], with a MAT of 17.7 °C, and MAP of 2514 mm [41]. (4) The Río Leona Formation and the associated fossil flora has given an age of 35–31 My [34], a MAT of 9.2 °C, and MAP of 820 mm [34]. (5) The Goterones–Matanzas flora outcrops within the Navidad Formation, dated to 22.1–18.9 My [57], with a MAT of 15.6 °C, and MAP of 1149 mm [41,58]. (6) The Cerro Los Pololos flora also belongs to the Navidad Formation, with an age of 12.8 My [57], a MAT of 17.7 °C, and MAP of 888 mm [58,59] (Table S5, Figure S5).

### 4.3. Evolution Models and Niche Conservatism

To evaluate phylogenetic niche conservatism, we compared the following models: (1) Brownian motion (BM), indicating a gradual and continuous drift; (2) Ornstein–Uhlenbeck model (OU) of stabilizing selection, a model with one or more optima; and (3) white noise (WN), which corresponds to random variation. Only BM and OU are indicators of niche conservatism through phylogeny [44]. This analysis was performed with the “fitContinuous” function [60] in R. Finally, the Akaike criterion was used to evaluate the best fit of the model.

The phylogenetic signal was evaluated using Pagel’s lambda parameter [31], which varies between 0 and 1, being 0 when there is no phylogenetic signal, which generally means that the character varies randomly in the phylogeny.

### 4.4. Ancestral Area Reconstruction

The BioGeoBEARS package (“BioGeography” with Bayesian Evolutionary Analysis) in R was used to estimate the most probable distribution area for each phylogenetic node. Six probabilistic models that operate under maximum likelihood were used: DEC (Dispersal—Extinction—Cladogenesis, [61]), DIVA (Dispersal—Vicariance Analysis, [62]), and BAYAREA [63]. In each case, a model was used that included the parameter *j*, which corresponds to the founder effect, that is, the emergence of a new distribution zone with respect to the most recent common ancestor [64]. The first corresponds mainly to a dispersion model, the second to vicariousness, and the third to sympatry [32]. The three models use two parameters: *d*, the dispersion rate; and *e*, the extinction rate.

## 5. Conclusions

Our study suggests that the MRCA of *Escallonia* would have been distributed in the central and southern zone of the Andes, above 30° of southern paleo-latitude, in mesothermal conditions during the Eocene. This is supported by the oldest fossil record of the genus (Argentine Patagonia) and the absence of mesothermal conditions in tropical latitudes.

Since the increase in the thermal gradient in the Ecuadorian polar region as a product of the global decline in temperatures at the Eocene/Oligocene boundary, *Escallonia* would have colonized new areas at lower latitudes such as the northern Andes, following its mesothermal niche, which was generated as the Andes rose. Thus, the new Andean habitats would have maintained the mesothermal conditions, functioning as a biological corridor for *Escallonia*.

The emergence of the Humboldt Current, the origin of the Atacama Desert and the marine transgressions of the Paranaense Sea would have influenced the east–west separation of the species and the colonization of Brazil by expanding the range of distribution from the central Andes. The microthermal condition would have been derived and would have evolved more recently (Oligocene) in species that inhabit temperate zones.

For the above reasons, *Escallonia* would not have originated in the tropical zone and migrated southwards, as proposed by TNH, but would have followed the opposite pattern, towards the tropics, as occurs with the genus *Nothofagus* [45].

## Figures and Tables

**Table 1:** Temperature (°C), precipitation (mm) and altitude (meters above sea level) minimum (min), maximum (max) and mean of each distribution zone of the genus *Escallonia*. The highest value mean of each climatic variable is highlighted in bold.

Bioclimatic Variable	Southern Andes			Central Andes			Northern Andes			Brazil		
	Min.	Max.	Mean	Min.	Max.	Mean	Min.	Max.	Mean	Min.	Max.	Mean
Mean annual temperature (Bio 1)	5.6	14.4	9.6	8.4	18.7	15.2	11.1	16.5	14.5	15.3	17.3	**16.5**
Temperature seasonality (Bio 4)	243.0	410.6	**359.4**	51.0	472.3	208.7	43.4	224.2	91.4	228.9	323.1	279.0
Mean temperature of wettest quarter (Bio 4)	3.3	12.8	6.1	7.0	22.2	16.9	11.3	18.6	15.1	17.7	20.0	**18.7**
Mean temperature of warmest quarter (Bio 10)	9.5	17.6	14.3	11.8	22.6	17.6	11.6	18.9	15.4	18.5	21.2	**19.9**
Mean temperature of coldest quarter (Bio 11)	1.4	11.5	5.1	4.9	15.9	12.3	10.1	15.8	**13.1**	11.8	13.4	12.8
Mean annual precipitation (Bio 12)	591.8	1852.3	1284.6	78.8	1372.9	807.9	871.2	1674.3	1185.1	1484.5	1809.1	**1584.9**
Precipitation of the wettest quarter (Bio 16)	345.6	925.7	**628.7**	51.5	516.8	375.5	436.9	642.4	526.6	479.7	750.1	549.1
Precipitation of the warmest quarter (Bio 18)	29	357.9	126	21.9	437.7	309.7	211.5	464.1	346.4	449.1	714.1	**529**
Precipitation of the coldest quarter (Bio 19)	328.6	866.8	**580.2**	26.8	440.2	94	49.6	369.7	219.6	219.7	354.1	283.8
Altitude (m)	239.4	1585.8	890.5	855	2811.2	2059.8	2132.9	3162.5	**2531.2**	678.6	1284.9	921.3

**Table 2:** Results of the analysis of phylogenetic niche conservation for each bioclimatic variable studied, and the respective value of each evolutionary model: Brownian (BM), Ornsted–Uhlembeck (OU), and random (WN) models; the highest values for each variable are indicated in bold. The results obtained from the phylogenetic signal using Pagel’s lambda [31] are shown for each bioclimatic variable studied. Variables with phylogenetic signal are highlighted in bold. The value of significance *p*-value is shown compared to 0 and 1.

Variable	BM	OU	WN	Lambda	*p*-Value (Different from 1)	*p*-Value (Different from 0)
**Bio 1**	**0.68**	0.32	<0.01	1	**1**	**<0.01**
**Bio 4**	**0.73**	0.27	<0.01	1	**1**	**<0.01**
**Bio 8**	**0.77**	0.23	<0.01	1	**1**	**<0.01**
**Bio 10**	0.15	**0.84**	<0.01	0.95	**1**	**<0.01**
**Bio 11**	**0.71**	0.29	<0.01	0.97	**1**	**<0.01**
**Bio 12**	0.04	**0.65**	0.31	0	<0.01	1
**Bio 16**	0.08	**0.58**	0.33	0	<0.01	1
**Bio 18**	**0.56**	0.44	<0.01	1	**1**	**<0.01**
**Bio 19**	**0.72**	0.28	<0.01	1	**1**	**<0.01**

## Data Availability

The data presented in this study are available on GBIF (https://www.gbif.org/, accessed on 15 November 2015), and Genbank (https://www.ncbi.nlm.nih.gov/genbank/, accessed on 5 May 2016).

## Supplementary Materials

The following supporting information can be downloaded at: https://www.mdpi.com/article/10.3390/plants13010133/s1. Table S1: Voucher specimens of *Escallonia* genus and outgroups obtained by Zapata (2013): collection (the herbarium where it was deposited is indicated in parentheses), locality of collection, geographic coordinates in decimal degrees, and GenBank numbers of Nia gen. Table S2: Minimum, maximum and average height values in which each species inhabits, where S the South Andes; C, The Central Andes; B, Brazil; and N to the North and Central Andes. Table S3: Values obtained from the reconstruction of the ancestral climate for each node in the phylogeny. Table S4: Weighted PNO values for each species in the nine bioclimatic variables analyzed. Table S5: Paleoclimatic estimates of mean annual temperature and annual precipitation for fossil localities in Chile and Argentina. Table S6: Results from ancestral area reconstruction models. The value of the best-fitting model, according to the value of LnL and weighted AIC (AIC wt), are indicated in bold. Figure S1: Comparison between the foliar areas of the species according to their geographical area, and from length and width data obtained from literature and digital herbaria, such as: Neotropical Herbarium Specimens, Herbario Virtual Austral Americano, SEINet: Arizona—New Mexico Chapter, Cooperative Taxonomic Resource for American Myrtaceae, Natural History Museum. Figure S2: Number assigned to each node of the phylogenetic reconstruction using Bayesian analysis. Figure S3: Reconstruction of the climatic niche of (a) Bio 4, (b) Bio 8, (c) Bio 10, (d) Bio 11, for the genus *Escallonia* over time. Figure S4: Reconstruction of the climatic niche of (a) Bio 16, (b) Bio 18, (c) Bio 19, for the genus *Escallonia* over time. Figure S5: Distribution map of the six formations with paleoclimatic estimation.
