# Supplementary material for: Testing the Tropical Niche Conservatism Hypothesis: Climatic Niche Evolution of Escallonia Mutis ex L. F. (Escalloniaceae)
Source: Plants (Basel). 2024 Jan 3;13(1):133. doi: 10.3390/plants13010133 (PMC10781032; doi:10.3390/plants13010133)
Supplement: Supplementary file 1 [file plants-13-00133-s001.zip › S_Tables.pdf]

Table S1: Voucher specimens of *Escallonia* genus and outgroups obtained by Zapata (2013): collection (the herbarium where it was deposited is indicated in parentheses), locality of collection, geographic coordinates in decimal degrees, and GenBank numbers of Nia gen.

**Especie**	**Código**	**Localidad**	**Latitud**	**Longitud**	**Gen Nia**
E. alpina	FZ331 (MO, CONC)	Santiago, Chile	-33.30	-70.32	KC355564
E. angustifolia	FZ324 (MO, HUSA)	Arequipa, Perú	-16.56	-71.45	KC355566
E. bifida	WP169 (MO, SPF)	Minas Gerais, Brasil	-22.49	-45.08	KC355572
E. callcottiae	FZ127A (MO)	Valparaíso, Chile	-33.64	-78.83	KC355575
E. cordobensis	JH3286 (MO, L, K)	Córdoba, Argentina	-30.85	-64.50	KC355576
E. discolor	FZ84 (ANDES)	Cundinamarca, Colombia	4.99	-74.15	KC355578
E. farinacea	WP152 (MO, SPF)	Sao Paulo, Brasil	-22.76	-45.55	KC355583
E. florida	FZ431 (M0, CONC)	Araucanía, Chile	-38.58	-71.63	KC355584
E. gayana	PB904 (E, CONC)	Araucanía, Chile	-38.47	-71.72	KC355587
E. herrerae	FZ190 (MO, MOL)	Cuzco, Perú	-13.47	-72.50	KC355588
E. hypoglauca	FZ304 (MO, LPB)	Santa Cruz, Bolivia	-17.83	-64.72	KC355590
E. illinita	FZ127 (MO, CONC)	Santiago, Chile	-33.01	-70.90	KC355595
E. laevis	LF87 (MO, SPF)	Paraná, Brasil	-25.24	-48.83	KC355599
E. ledifolia	LF59 (MO, SPF)	Santa Catarina, Brasil	-27.84	-49.65	KC355601
E. leucantha	FZ383 (MO, CONC)	Bío-Bío, Chile	-37.81	-73.14	KC355605
E. megapotamica	LF72 (MO, SPF)	Santa Catarina, Brasil	-26.10	-49.83	KC355608
E. micrantha	FZ242 (M0, MOL)	Cajamarca, Perú	-7.08	-79.05	KC355610
E. millegrana	FZ289 (MO, LPB)	Cochabamba, Bolivia	-17.84	-65.46	KC355611
E. myrtilloides	BH23603 (INB)	San José, Costa Rica	9.60	-83.83	KC355613
E. myrtoidea	FZ497 (MO, CONC)	Maule, Chile	-35.92	-71.37	KC355620
E. paniculata	FZ245 (MO, MOL)	Piura, Perú	-5.37	-79.58	KC355623
E. pendula	FZ206 (MO, MOL)	Cajamarca, Perú	-6.87	-78.11	KC355626
E. petrophila	LF44 (MO, SPF)	Santa Catarina, Brasil	-28.06	-49.37	KC355630
E. piurensis	FZ239 (MO, MOL)	Cajamarca, Perú	-7.33	-78.81	KC355631
E. polifolia	FZ224 (MO, MOL)	Amazonas, Perú	-6.71	-77.85	KC355632
E. pulverulenta	FZ361 (MO, CONC)	Bío-Bío, Chile	´-37.69	-72.73	KC355636
E. resinosa	FZ182 (MO, MOL)	Cuzco, Perú	-13.18	-72.29	KC355637
E. reticulata	AL360 (MO)	Chuquisaca, Bolivia	-19.81	-63.72	KC355640
E. revoluta	FZ359 (MO, CONC)	Bío-Bío, Chile	-37.64	-72.79	KC355643
E. rosea	FZ531 (MO, CONC)	Los Ríos, Chile	-40.18	-73.44	KC355652
E. rubra	FZ406 (MO, CONC)	Bío-Bío, Chile	-37.39	-71.46	KC355656
E.schreiteri	NL58 (MO)	Santa Cruz, Bolivia	-17.86	-64.63	KC355658
E. serrata	KFC1662A (MO)	Tierra del Fuego, Argentina	-54.94	-66.93	KC355660
E. tucumanensis	FZ10377C (MO, SI)	Salta, Argentina	-22.33	-64.72	KC355663
E. virgata	FZ370 (MO, CONC)	Bío-Bío, Chile	-37.81	-73.02	KC355664
Forgesia racemosa	JF425 (REU)	Eden, Réunion	-21.00	55.63	KC355671
Valdivia gayana	FZ99 (MO, CONC)	Los Lagos, Chile	-39.88	-73.42	KC355672

Table S2: Minimum, maximum and average height values in which each species inhabits, where S the South Andes; C, The Central Andes; B, Brazil; and N to the North and Central Andes.

**Especie**	**Altura mínima**	**Altura máxima**	**Altura promedio**	**Zona**
E. alpina	31	2787	1419.3	S
E. angustifolia	1130	3940	2481.3	C
E. bífida	7	1863	1008.7	B
E. callcottiae	5	428	239.4	S
E. cordobensis	298	2005	855	C
E. discolor	203	3840	2449.6	N
E. farinacea	603	1897	968.7	B
E. florida	157	2251	1091.7	S
E. gayana	47	1184	561.6	S
E. herrerae	125	3949	2811.2	C
E. hypoglauca	203	3470	2260.5	C
E. illinita	61	3694	1459.4	S
E. laevis	9	2503	1284.9	B
E. ledifolia	768	1639	1047.2	B
E. leucantha	27	1824	588.7	S
E. megapotamica	2	1318	678.6	B
E. micrantha	196	3406	2427.9	N, C
E. millegrana	146	2865	1669.2	C
E. myrtilloides	27	4816	3029.5	N, C
E. myrtoidea	225	3396	1585.8	S
E. paniculata	90	3482	2132.9	N, C
E. pendula	159	4209	2241.6	N, C
E. petrophila	2	1415	766.2	B
E. piurensis	1588	3036	2328.9	C
E. polifolia	1938	3576	2709.3	C
E. pulverulenta	7	1824	585.3	S
E. resinosa	203	4733	3162.5	N, C
E. reticulata	614	2385	1654.7	C
E. revoluta	3	1827	713.9	S
E. rosea	27	1705	1000.2	S
E. rubra	0	1824	687.5	S
E. schreiteri	1171	3532	2193	N, C
E. serrata	7	1824	540.7	S
E. tucumanensis	387	2063	1378.1	C
E. virgata	4	2291	1102.6	S

Table S3: Values obtained from the reconstruction of the ancestral climate for each node in the phylogeny.

Node	Bio1	Bio4	Bio8	Bio10	Bio11	Bio12	Bio16	Bio18	Bio19
**1**	13.9	210.4	12.8	16.2	10.8	1095.4	510.5	254.8	304.7
**2**	14.5	215.7	12.6	16.0	10.5	1147.1	525.7	268.5	319.8
**3**	14.5	304.0	8.2	14.4	6.6	1250.3	581.7	191.7	475.6
**4**	15.8	316.3	7.6	14.4	6.2	1296.0	605.5	179.9	515.5
**5**	14.9	322.4	7.2	14.1	5.7	1274.2	587.7	175.6	506.1
**6**	13.3	324.8	7.5	14.6	6.2	1190.0	571.2	143.9	493.5
**7**	11.0	358.2	6.2	14.4	5.1	1165.4	577.2	119.1	523.0
**8**	11.0	389.7	4.9	14.0	3.9	1006.8	507.9	95.3	469.7
**9**	12.5	360.1	6.0	14.7	5.3	1299.5	652.7	118.1	605.9
**10**	12.5	329.7	5.9	12.5	3.9	1399.0	585.3	230.4	512.4
**11**	14.3	306.4	8.0	14.3	6.4	1221.7	567.2	190.6	461.7
**12**	14.4	293.2	8.6	14.4	6.9	1207.6	557.0	203.2	437.5
**13**	14.4	251.6	10.5	14.7	8.3	1091.2	478.5	240.2	319.6
**14**	16.3	294.4	11.3	15.6	8.1	928.5	404.9	224.6	262.5
**15**	16.0	291.1	14.9	17.0	9.6	942.0	369.4	304.5	182.2
**16**	16.3	296.7	17.3	18.8	11.2	1155.4	437.2	390.6	201.1
**17**	16.3	297.8	18.3	19.7	12.1	1358.7	472.1	439.4	253.4
**18**	16.3	313.7	19.3	21.1	13.1	1426.1	484.6	455.5	266.6
**19**	17.0	300.0	18.4	19.4	11.8	1163.0	462.0	418.6	176.0
**20**	12.8	324.9	19.3	20.0	11.7	973.7	430.9	390.7	110.5
**21**	9.5	388.2	20.6	21.1	11.1	783.2	373.0	351.2	59.1
**22**	9.5	267.7	18.6	19.6	12.7	1458.1	545.8	502.5	236.5
**23**	9.1	260.7	18.0	19.3	12.4	1557.9	554.4	514.0	274.0
**24**	10.0	124.8	12.1	13.7	10.6	1160.7	471.4	319.1	228.4
**25**	10.0	350.2	6.0	14.1	5.1	1412.0	693.3	154.4	624.9
**26**	8.7	174.1	14.8	16.6	12.2	1121.4	505.2	313.7	249.5
**27**	10.6	157.3	15.4	16.5	12.6	1067.0	488.6	322.8	200.2
**28**	10.2	104.9	14.6	15.3	12.7	1058.3	466.8	299.5	217.1
**29**	10.6	104.9	13.8	14.1	11.5	853.2	403.2	305.7	105.0
**30**	14.7	137.0	16.9	17.7	14.3	1164.5	499.6	367.9	217.9
**31**	15.0	206.4	13.0	16.3	11.1	1056.6	499.1	244.5	293.4
**32**	14.5	153.9	14.5	16.1	12.3	1027.7	468.0	289.8	210.4
**33**	13.4	96.1	14.6	15.3	13.0	1052.3	456.9	304.0	181.1
**34**	16.3	159.1	16.0	16.8	12.8	974.2	448.0	320.9	156.9

Table S4: Weighted PNO values for each species in the 9 bioclimatic variables analyzed.

Specie	Bio 1	Bio 4	Bio 8	Bio 10	Bio 11	Bio 12	Bio 16	Bio 18	Bio 19
E. alpina	7.1	398.5	3.5	12.3	2.0	1038.1	479.9	123.9	447.7
E. angustifolia	8.4	265.5	8.9	11.4	4.6	101.8	62.6	40.1	26.7
E. bifida	17.0	295.8	17.7	20.6	13.3	1484.5	508.5	487.3	271.1
E. callcottiae	13.9	231.8	12.3	16.9	11.0	1011.4	503.5	123.1	367.3
E. cordobensis	15.7	471.4	21.2	21.7	9.5	642.5	297.3	291.4	34.8
E. discolor	15.8	52.3	15.6	16.4	15.1	1459.7	572.4	263.8	458.4
E. farinacea	17.4	249.5	19.9	20.3	14.0	1553.8	612.3	563.2	222.1
E. florida	9.1	383.9	4.8	14.2	4.3	1407.2	734.7	105.4	690.4
E. gayana	10.6	347.7	6.9	15.2	6.3	1633.7	813.1	142.8	761.8
E. herrerae	14.1	81.1	13.5	13.8	11.9	960.8	431.8	307.7	95.7
E. hypoglauca	16.3	247.9	18.5	19.0	12.8	976.6	484.6	414.1	82.5
E. illinita	9.8	386.7	5.6	14.7	4.7	575.9	328.5	33.4	308.6
E. laevis	16.0	232.0	17.8	18.7	12.3	1588.2	630.2	571.4	225.3
E. ledifolia	15.3	268.2	18.3	18.6	11.8	1630.2	516.6	504.6	331.4
E. leucantha	9.4	356.5	5.4	14.0	4.7	1506.7	734.2	141.6	692.7
E. megapotamica	17.1	324.6	19.5	21.6	13.3	1506.0	485.1	456.4	298.5
E. micrantha	14.7	52.9	13.0	13.3	12.1	1041.0	435.4	299.6	202.8
E. millegrana	17.9	270.6	20.5	20.9	14.1	853.8	440.6	373.4	57.3
E. myrtilloides	12.7	69.7	12.6	12.9	11.4	1402.7	552.0	402.1	260.7
E. myrtoidea	9.8	412.3	5.2	15.2	4.5	816.9	466.5	42.9	438.5
E. paniculata	16.7	52.6	16.5	16.9	15.8	1624.4	617.2	441.2	342.3
E. pendula	16.0	53.4	15.7	16.1	14.8	1168.5	470.9	314.5	237.2
E. petrophila	16.1	282.3	17.7	19.6	12.3	1627.4	487.3	468.2	360.2
E. piurensis	14.2	80.2	14.8	15.0	12.9	700.7	327.7	291.0	53.1
E. polifolia	13.0	53.1	13.3	13.5	12.2	988.2	383.5	314.9	104.8
E. pulverulenta	12.6	352.1	8.8	17.2	8.2	1027.0	558.2	77.7	508.1
E. resinosa	11.8	129.7	11.8	12.1	9.0	800.6	415.0	326.6	44.6
E. reticulata	17.7	184.4	19.5	19.6	15.0	747.8	376.3	348.9	61.9
E. revoluta	11.6	365.6	6.5	15.8	5.9	1226.4	646.5	93.7	601.9
E. rosea	8.8	373.6	4.6	13.8	4.1	1621.4	788.2	154.7	746.8
E. rubra	9.7	355.9	5.4	14.0	4.6	1511.4	690.5	176.0	647.1
E. schreiteri	16.4	228.5	18.4	18.9	13.1	975.4	498.9	387.5	67.8
E. serrata	5.5	310.9	5.0	9.4	1.4	1411.2	477.6	339.5	384.0
E. tucumanensis	17.3	368.3	21.2	21.6	12.2	733.3	390.8	371.3	32.0
E. virgata	7.8	384.1	4.2	12.7	2.9	1149.6	546.4	122.4	513.5

Table S5: Paleoclimatic estimates of mean annual temperature and annual precipitation for fossil localities in Chile and Argentina.

Locality	Country	Age (My)	Mean anual temperature (°C)	Anual precipitation (mm)
Laguna del Hunco	Patagonia, Argentina	52 (Wilf et al., 2003)	17.2 (±2.3) (Wilf et al., 2003)	1673 (± 426) (Wilf et al., 2003)
Quinamávida	VII - IX Región, Chile	46 – 45 (Flynn et al., 2008)	18.3 (±2.1) (Hinojosa, 2005)	888 (± 426) (Hinojosa, 2005)
Río Turbio	Patagonia, Argentina	44.6 – 34 (Guerstein et al. 2010)	17.7 (±2.1) (Hinojosa, 2005)	2514 (± 426) (Hinojosa, 2005)
Río Leona	Patagonia, Chile	35 – 31 (Gutiérrez, 2017)	9.2 (±2.1) (Gutiérrez, 2017)	820 (± 470) (Gutiérrez, 2017)
Navidad – Goterones-Matanza	V Región, Chile	22.1 – 18.9 (Gutiérrez et al., 2013)	15.6 (±2.4) (Hinojosa, 2005)	1149 (+136/-122) (Hinojosa, 2005; Hinojosa et al., 2015)
Navidad – Cerro Los Pololos	VI Región, Chile	12.8 (Gutiérrez et al., 2013)	17.7 (±2.8) (Pino, 2016; Hinojosa, 2015)	888 (+349/-576) (Pino, 2016; Hinojosa, 2015)

Table S6: Results from ancestral area reconstruction models. The value of the best-fitting model, according to the value of LnL and weighted AIC (AIC wt), are indicated in bold.

**Model**	**LnL**	**Num. parameters**	**d (dispersion)**	**e (extinction)**	**j (jumps)**	**AIC**	**AIC wt**
DEC	**-49.48**	2	0.0084	1.00E-12	0	103	**0.65**
DEC+J	-49.13	3	0.0073	1.00E-12	0.02	104.3	0.34
DIVALIKE	-56.04	2	0.01	1.00E-12	0	116.1	0.0009
DIVALIKE+J	-53.09	3	0.0078	1.00E-12	0.021	112.2	0.0065
BAYAREALIKE	-62.54	2	0.011	0.025	0	129.1	1.40E-06
BAYAREALIKE+J	-55.09	3	0.0049	0.0084	0.031	116.2	0.0009
