# Supplementary figures and images for: Testing the Tropical Niche Conservatism Hypothesis: Climatic Niche Evolution of Escallonia Mutis ex L. F. (Escalloniaceae)
Source: Plants (Basel). 2024 Jan 3;13(1):133. doi: 10.3390/plants13010133 (PMC10781032; doi:10.3390/plants13010133)

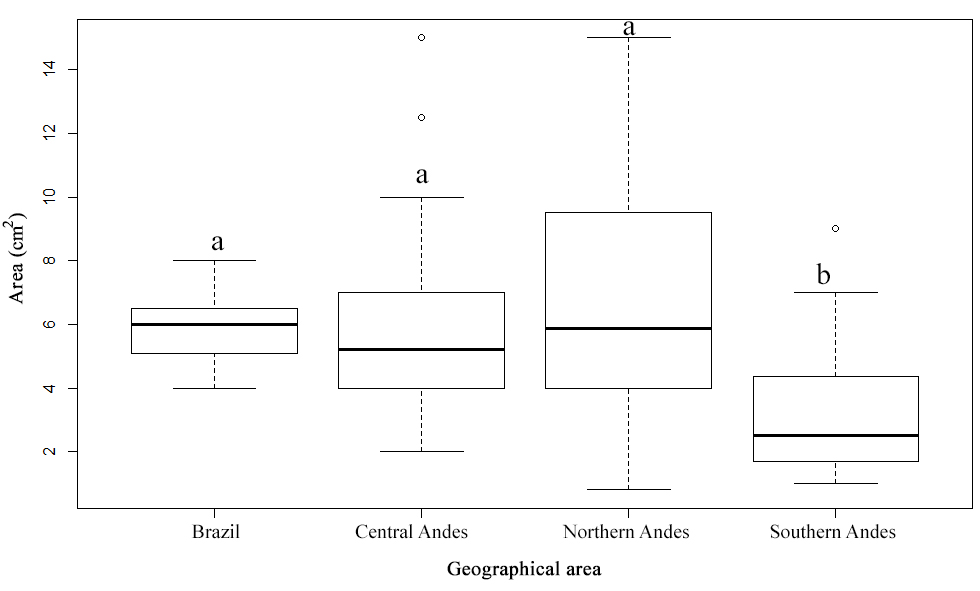

Supplement: Supplementary file 1 [file plants-13-00133-s001.zip › Figure_S1.jpg]

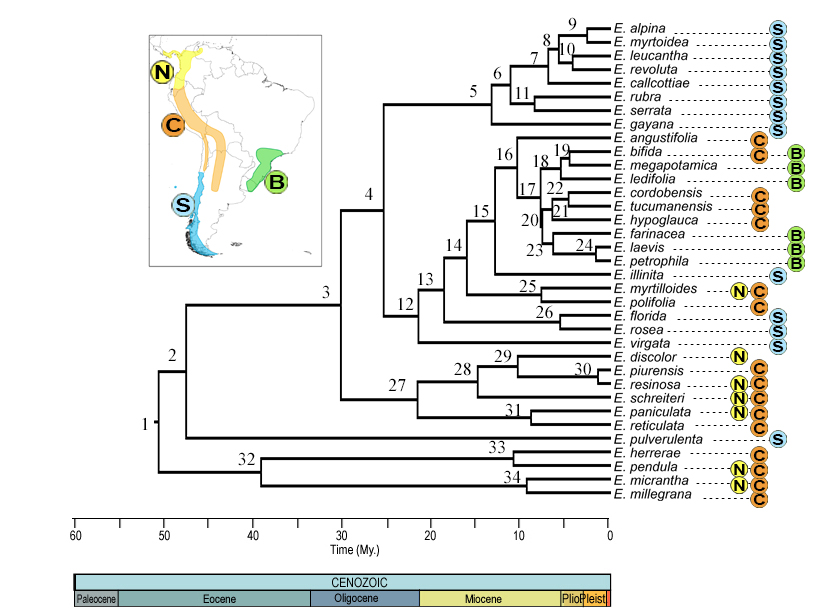

Supplement: Supplementary file 1 [file plants-13-00133-s001.zip › Figure_S2.jpg]

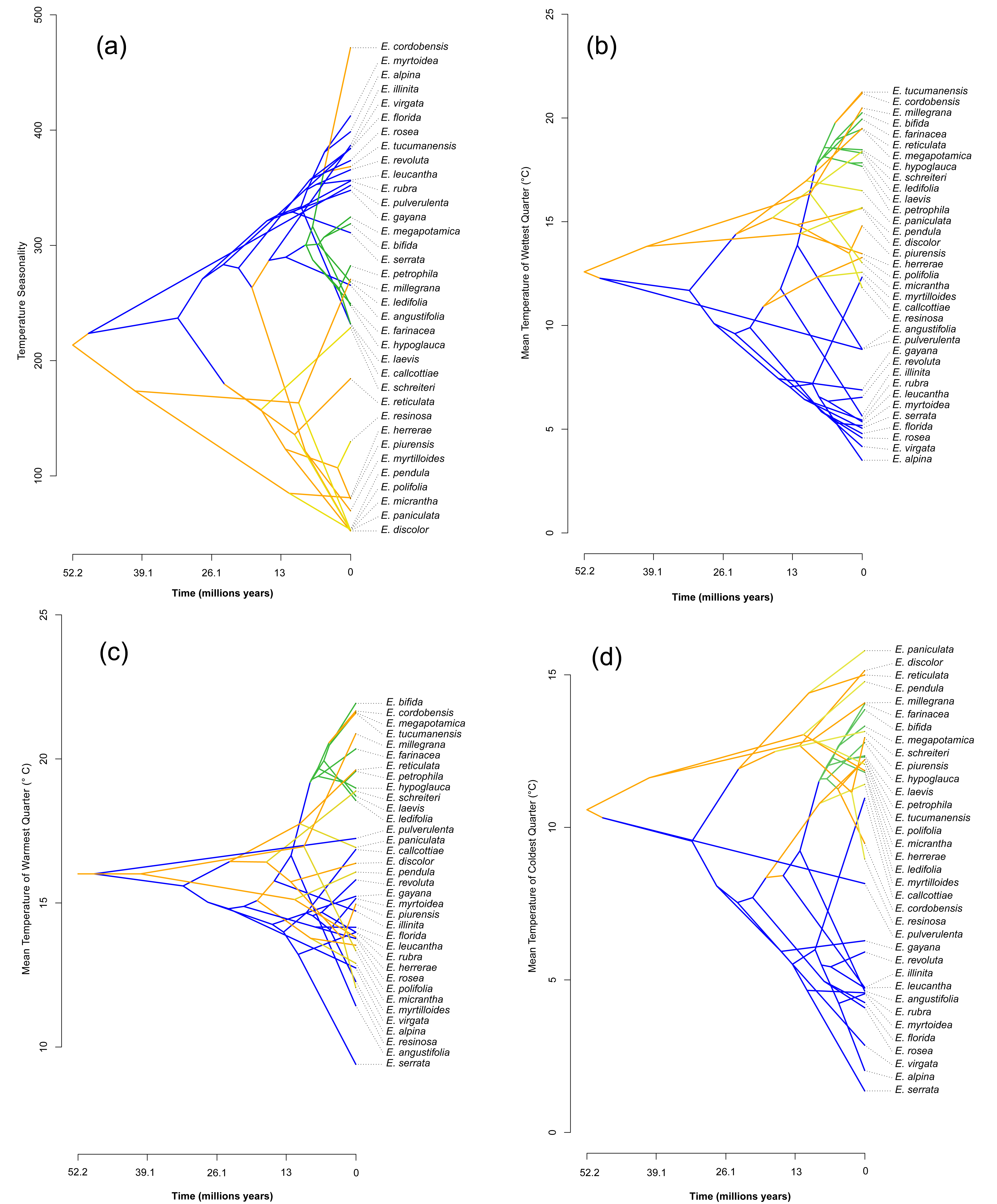

Supplement: Supplementary file 1 [file plants-13-00133-s001.zip › Figure_S3.jpg]

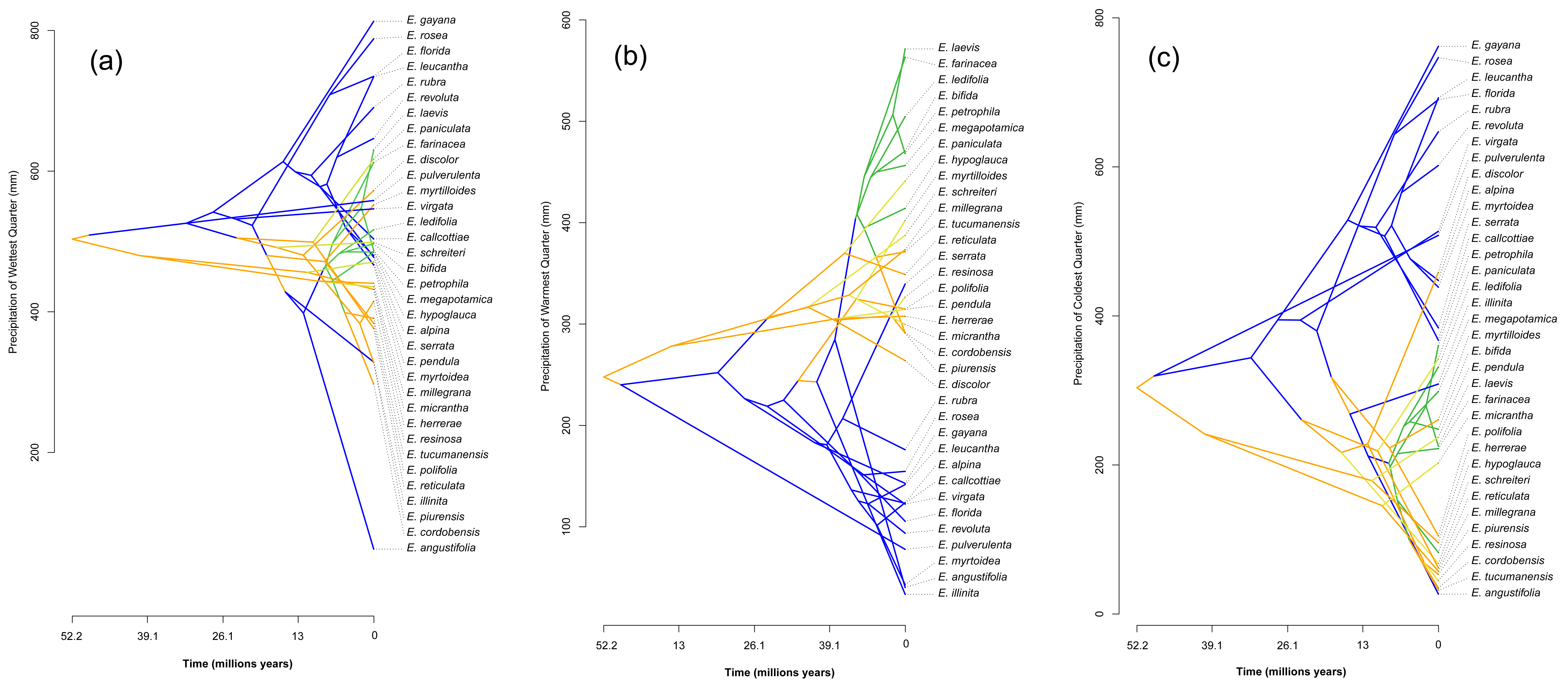

Supplement: Supplementary file 1 [file plants-13-00133-s001.zip › Figure_S4.jpg]

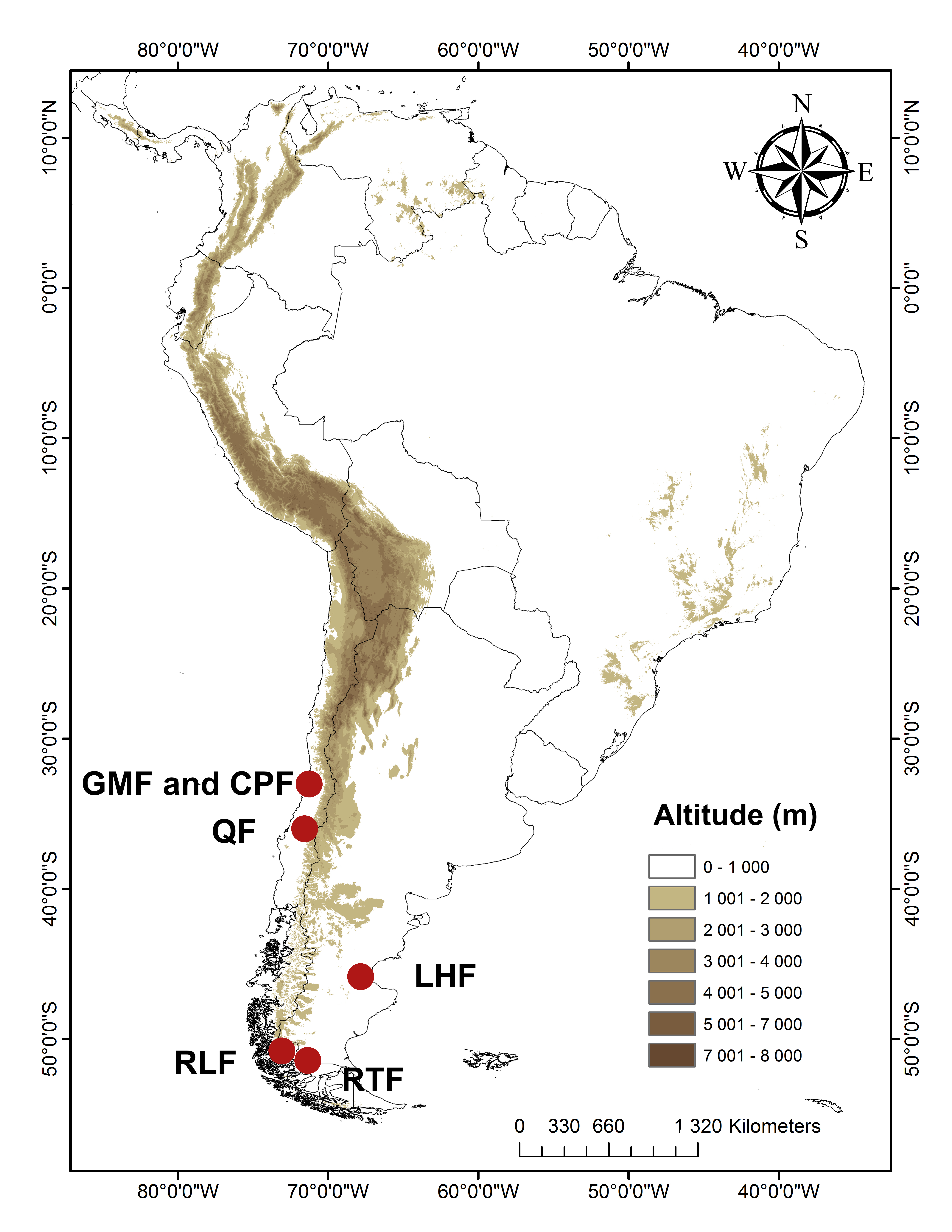

Supplement: Supplementary file 1 [file plants-13-00133-s001.zip › Figure_S5.jpg]
